# Nutzen und Risiken psychiatrischen Handelns und das Selbstbestimmungsrecht des Patienten

**DOI:** 10.1007/s00115-020-01055-z

**Published:** 2021-01-22

**Authors:** Hanfried Helmchen

**Affiliations:** Klinik für Psychiatrie und Psychotherapie, Charite – Universitätsmedizin Berlin, Berlin, Deutschland

**Keywords:** Wahrnehmung von Risiken, Akzeptanz des Selbstbestimmungsrechtes, Nutzen-Risiko-Verhältnis, Entwicklung versorgender Interventionen, Entwicklung therapeutischer Interventionen, Recognition of risks, Acceptance of self-determination, Benefit-risk ratio, Development of caring interventions, Development of therapeutic interventions

## Abstract

Die Geschichte der Psychiatrie zeigt, dass ein Selbstbestimmungsrecht des psychisch Kranken im 19. Jahrhundert weitgehend unbekannt war, erst spät in der 2. Hälfte des 20. Jahrhunderts mit dem rechtlichen Konzept der Einwilligung nach Aufklärung, dem „informed consent“, in der Medizin bekannt wurde und seit Beginn des 21. Jahrhunderts in der medizinischen Praxis zunehmend wahrgenommen und respektiert wird; diese Wahrnehmungsänderung trägt zu einem Wandel von einer paternalistischen zu einer partizipativen ärztlichen Einstellung bei. Im Kontext einer emanzipatorischen Entwicklung der Gesellschaft nimmt mit den wachsenden Möglichkeiten wirksamer Therapien, die selten ohne Risiken sind, die Notwendigkeit zu, den Patienten über den intendierten Nutzen und potenzielle Risiken der empfohlenen Intervention zu informieren und ihm damit die Voraussetzung zu schaffen, sein Selbstbestimmungsrecht wahrzunehmen. Zudem wachsen mit dem Übergang von einer sehr erfolgreichen Akutmedizin mit allerdings oft nur kurzem Arzt-Patienten-Kontakt zur Langzeitmedizin chronischer Erkrankungen die Möglichkeiten, den Patienten, insbesondere den psychisch Kranken, seine individuellen Eigenheiten, seine Einschränkungen und Fähigkeiten besser wahrzunehmen, ihn als Individuum, als Menschen in seiner jeweiligen Eigenart zu erleben und sein Selbstbestimmungsrecht zu respektieren, indem wir ihm dabei helfen, Nutzen und Risiken einer empfohlenen Intervention zu verstehen und selbstbestimmt abzuwägen.

## Abstract (en)

The history of psychiatry shows that a right of self-determination of the mentally ill was widely unknown in the nineteenth century and became known in medicine through the juridical concept of informed consent as late as in the second half of the twentieth century. Since the beginning of the twenty-first century this human right has been increasingly recognized and respected in medical practice. This change of recognition is contributing to a change from a paternalistic to a participative medical attitude. In the context of an emancipatory development of society the increasing possibilities of effective therapies, which are rarely without risks, stimulate the necessity to inform the patient about the intended benefits and the potential risks of the recommended intervention. This gives the patient the opportunity to exercise the right of self-determination. Furthermore, by the transition from very successful acute medicine, although often with only short-term contact between physician and patient to long-term therapies of chronic diseases, the possibilities to understand the patient are increased, particularly in the mentally ill patient. This also enables the individual characteristics to be recognized better, both the restrictions and capabilities, to experience the patient as an individual, as a human being with individual peculiarities and to respect the right of self-determination by helping the patient to understand the benefits and risks of a recommended intervention and to balance them in a self-determined mode.

## Hintergrund

Psychiater handeln mit der Intention, ihren Patienten zu nützen. Dabei stoßen sie auf Begrenzungen; zwei der wichtigsten sind zum einen die Wahrnehmung von Risiken und zum anderen die Akzeptanz des Selbstbestimmungsrechtes des Patienten.

Interventionen werden eingeführt, wenn sie Nutzen für den Patienten versprechen. Waren es früher Interventionen, die aus Überzeugungen – etwa humanistischen Ideen – abgeleitet oder mit kasuistischen Beobachtungen begründet wurden, sind es heute evidenzbasierte Verfahren, deren Wirksamkeit (Nutzen) und Sicherheit (Risiken) in kontrollierten Versuchen belegt sein müssen. Vor jeder Anwendung muss das Verhältnis von intendiertem Nutzen zu potenziellen Risiken sowohl für den Psychiater wie auch für den Patienten vertretbar sein und vom Patienten akzeptiert werden. Bei längerer Anwendung sowohl beim einzelnen Patienten wie auch in der breiten Anwendung bei vielen Patienten und unter den Bedingungen des Alltags (z. B. Phase IV der Arzneimittelprüfung) werden unerwünschte Wirkungen (z. B. der Pharmakotherapie) deutlicher und häufiger und damit wird das *Nutzen-Risiko-Verhältnis* ungünstiger. Eine Aufgabe des behandelnden Psychiaters ist es, diese Verschiebung wahrzunehmen und seine Intervention optimierend zu ändern, bevor sie der Patient von sich aus abbricht.

Damit kommt die *Selbstbestimmung* des Patienten ins Spiel. Ein Beispiel: In den 1950er-Jahren hörte ich als junger Arzt eher zufällig, dass Juristen jede ärztliche Intervention am Patienten als Körperverletzung werten, sofern sie nicht durch Einwilligung des Patienten legitimiert worden ist. Ich war empört, behandelte ich doch Patienten in deren „besten Interesse“ und sah die Einwilligung des hilfesuchenden Kranken als implizit gegeben an. Heute wird das Handeln bzw. der Handlungsrahmen des Arztes durch das Selbstbestimmungsrecht als einer wesentlichen Dimension des Arzt-Patienten-Verhältnisses ebenso begrenzt wie auch erweitert: Vor dem Hintergrund der seit 2009 auch hierzulande geltenden UN-Behindertenrechtskonvention (BRK) wird uns Ärzten die Bedeutung von Menschenrechten explizit und nachhaltig bewusst und wir respektieren das Selbstbestimmungsrecht des Patienten [1]. Es verändert das Arzt-Patienten-Verhältnis. Diese Veränderung erscheint als Wandel vom paternalistischen ärztlichen Handeln **für** den Patienten über eine partizipative Entscheidungsfindung **mit** dem Patienten zur autonomen Entscheidung **allein** des Patienten; solcher Einstellungswandel soll im Respekt vor dem Selbstbestimmungsrecht die Möglichkeiten des Patienten zur eigenständigen Entscheidung stärken [2]. Aber auch wenn ein Patient die Entscheidung für sich allein trifft, sollte der Arzt wenn irgend möglich sicherstellen, dass dem Patienten die Konsequenzen seiner Entscheidung klar sind.

Bei psychisch Kranken kann hier jedoch eine Grenze zu schädlichen Folgen überschritten werden, wenn der Respekt vor dem Selbstbestimmungsrecht des Patienten in Widerspruch zur Fürsorgepflicht des Arztes gerät; vor allem, wenn die Intensität einer psychischen Störung die Selbstbestimmungsfähigkeit des Kranken beeinträchtigt. Bei Wahnkranken, deren Wahn sie zu lebensgefährlichen Handlungen zwingt, oder bei dementen und deliranten Menschen, die in ihrer Verwirrtheit Hilfe gar nicht aufsuchen können, erscheint die Selbstbestimmungsfähigkeit aufgehoben und der Arzt muss deshalb für den Kranken handeln. Ist jedoch – wohl viel häufiger – die psychische Störung nicht so ausgeprägt, kann es schwierig sein festzustellen, ob und inwieweit die Selbstbestimmungsfähigkeit eingeschränkt ist. Aufgabe des Psychiaters ist es dann, dies zu prüfen wie auch den richtigen Ort zwischen diesen Entscheidungsoptionen zu finden: ein situationsangemessenes Verhalten, das einerseits so wenig paternalistisch wie im ausschließlichen Interesse des Patienten unvermeidbar nötig ist und andererseits dessen Selbstbestimmungsrecht so weit wie möglich respektiert. Dies bedeutet auch, die Selbstbestimmungsfähigkeit des psychisch Kranken zu stärken, indem seine Fähigkeiten gesucht und unterstützt werden.

Vor diesem Hintergrund sollen einige Entwicklungsschritte der psychiatrischen Versorgung wie auch der psychiatrischen Therapie verdeutlichen, dass nützliche psychiatrische Interventionen auch Risiken enthalten und sogar gefährlich werden können, vor allem dann, wenn sie unzureichend umgesetzt oder dogmatisch verabsolutiert werden. Wahrnehmung von Risiken ebenso wie wachsende Verbesserung von Versorgung und Behandlung öffnen auch Spielräume für den Einfluss des Selbstbestimmungsrechts nicht zuletzt auf den Umgang mit Risiken.

## Entwicklung versorgender Interventionen

### Anerkennung als psychisch krank vs. Asylierung

So begann mit der Aufklärung im 18. Jahrhundert insofern eine positive Entwicklung, als psychisch Kranke nicht mehr als verhexte oder von Dämonen besetzte Menschen angesehen, sondern als Kranke anerkannt und durch Unterbringung in Asylen geschützt wurden, wenn beispielsweise Angehörige sich gegen ausgeprägte Verhaltensstörungen ihrer psychisch Kranken offenbar nicht anders zu helfen wussten, als diese zu fesseln oder in Tierställen wegzusperren [3] oder sie in ein Irrenasyl zu bringen [4]. Meist kommunale oder staatliche Irrenasyle waren Ausdruck „der besondern Aufsicht und Vorsorge des Staats“ wie es im preußischen Landrecht von 1794 heißt [5].^1^ Allerdings stand dabei der Sicherungsaspekt oft ganz im Vordergrund staatlichen Handelns, während die in den Asylen tätigen Ärzte über therapeutisch ungeeignete Räumlichkeiten und unzureichend ausgebildetes Personal nachhaltig klagten … Als eine Stimme von vielen sei die von Kraepelin zitiert:

Dieselben Irrenärzte, die man bisweilen in merkwürdiger Verkennung der geschichtlichen Entwicklung gewissermassen als geborene Feinde der Kranken und Gesunden zu brandmarken beliebt, sind es gewesen, die in mühseliger, aufopferungsreicher Berufsarbeit ihren Pflegebefohlenen die Ketten gelöst haben, in welche sie Roheit und Unkenntnis so lange geschmiedet hatte [6, S. 630].

Die Klage hält bis heute an, denn inzwischen hat die Psychiatrie zwar ein breites Arsenal therapeutisch wirksamer Verfahren entwickelt, kann sie aber oft nicht zur vollen Wirkung bringen, weil die Rahmenbedingungen unzureichend sind. Die zentrale Schwäche der Rahmenbedingungen ist die Zeitnot infolge Personalmangels. Sie wird besonders evident durch den erfreulichen Zuwachs an Methoden der Psychotherapie, der rehabilitativen Unterstützung und Zuwendung, also Methoden, die auf dem Gespräch basieren und der Zeit bedürfen; Zeitmangel behindert auch Gespräche zur Stärkung der Selbstbestimmungsfähigkeit des Patienten und die Teilnahme der psychiatrischen Mitarbeiter an qualifizierender Weiterbildung zur ethischen und rechtlichen Sensibilisierung. Diese Entwicklung zum differenzierteren Umgang mit psychisch Kranken hat auch rechtlichen Niederschlag gefunden. Aber bereits 2014 musste die Deutsche Gesellschaft für Psychiatrie und Psychotherapie, Psychosomatik und Nervenheilkunde (DGPPN) warnend feststellen:

Eine Anhebung der rechtlichen, ethischen und professionellen Standards ohne gleichzeitige strukturelle und finanzielle Realisierungsmöglichkeit ist unehrlich, frustriert die Beteiligten und trägt nicht zu einer besseren Patientenversorgung bei. Denn die gestiegenen rechtlichen Anforderungen im Betreuungsrecht (BGB) stehen den zeitgleich gestiegenen Kosteneffizienzanforderungen im Rahmen des neuen pauschalierenden Entgeltsystems für Psychiatrie und Psychosomatik (PEPP) im Rahmen des Sozialrechts entgegen. Wenn die Spannung zwischen Anforderungen des BGB und unzureichender Ausstattung durch das Sozialrecht weiter zunehmen, wird die Gesetzgebung unglaubwürdig und die im Gesundheitswesen Tätigen kommen in eine ethisch nicht zu verantwortende Konfliktsituation [7].

### Heilbarkeit vs. Unheilbarkeit

Da es seit dem 19. Jahrhundert an zureichender Ausstattung sowie an wirksamer Behandlung mangelte, sammelten sich chronisch psychisch Kranke als Pflegefälle in den Anstalten an, die dadurch oft zu reinen Verwahranstalten verkamen. Das Dogma der Unheilbarkeit verbesserte zwar durch Konzentration der begrenzten Ressourcen die Lebens- und Behandlungsbedingungen der als heilbar angesehenen Patienten, lieferte jedoch die „Unheilbaren“ einem langen bis lebenslangen Aufenthalt in Pflegeanstalten aus. Der Versuch Wilhelm Griesingers, diese in praxi unhaltbare Unterscheidung durch eine realitätsnähere Unterscheidung in akute und chronische Krankheitszustände zu ersetzen, scheiterte mit dem frühen Tode Griesingers, und die Erkenntnis Esquirols auch sozialer Einflüsse auf das Erscheinungsbild psychischer Krankheit war nicht stark genug, die manchenorts zum „sozialen Tod“ führende dauerhafte Isolierung psychisch Kranker in reinen Pflegeanstalten zu vermeiden. Die Folge war therapeutische Resignation; sie wurde noch unterstützt durch den „therapeutischen Nihilismus“, den manche akademischen Psychiater verbreiteten, die ihre Aufgabe darin sahen, die Ursachen der Geisteskrankheiten aufzuklären, nicht aber, die Kranken zu behandeln [8].

### Krankheit vs. Kranksein

Damit wird auch das Risiko angesprochen, dass die Annäherung der Psychiatrie an die Medizin, d. h. die Übernahme des „medizinischen Modells“, den Kranken nicht nur Schutz und Behandlung bot. Psychiater, die sich um die vorige Jahrhundertwende auf die Erkennung und Behandlung von Krankheit fokussierten, folgten dem somatischen Krankheitskonzept der akademisierten Psychiatrie, das mittels naturwissenschaftlicher Methoden auf die Erkennung der im Gehirn vermuteten Bedingungen der psychopathologischen Krankheitserscheinungen zielte. Dies ebenso wie die im letzten halben Jahrhundert gewachsene standardisierte Erfassung psychopathologischer Symptome und deren operationalisierte Zuordnung zu vorgegebenen (und fälschlicherweise reifizierten) Kategorien engte den Blick klinisch tätiger Psychiater ein auf die Erkennung und Behandlung von *Krankheit*, sodass sie das Ganze des Patienten, sein *Kranksein,* aus dem Auge verlieren konnten, sein Erleben von Krankheit und seine Bewältigungsbemühungen, auch sein Scheitern in einem spezifischen sozialen Kontext [9]. Aber gerade im Umgang des Kranken mit seiner Störung lassen sich Ansatzpunkte für eine Stärkung der Selbstbestimmungsfähigkeit finden. Bereits vor mehr als 100 Jahren hat Karl Jaspers beide Perspektiven, die des Erklärens objektivierbarer Sachverhalte und die des Verstehens subjektiver Gegebenheiten, als komplementär und unverzichtbar für die Tätigkeit des Psychiaters beschrieben und damit jeder Verabsolutierung von Partialerkenntnissen eine Absage erteilt.

### Behandlung vs. Verwahrung

Ein psychiatriehistorischer Blick zurück ins 19. Jahrhundert lässt also erkennen, dass sich der Nutzen für psychisch Kranke, als Kranke anerkannt zu werden und den Schutz des medizinischen Modells (heute bis hin zur Krankenversicherung) zu erhalten, infolge unzureichender Rahmenbedingungen psychiatrischer Versorgung zum Schaden der psychisch Kranken wandelte, wenn sie in heruntergekommen Irrenasylen als chronische Pflegefälle mit Hospitalismus nur noch verwahrt – und schließlich im Nationalsozialismus als „nutzlose Esser“ umgebracht wurden. Diese unmenschliche Konsequenz der nationalsozialistischen Psychiatriepolitik des „Heilens und Vernichtens“ psychisch Kranker zeigt die Gefahren einer Ideologie, die auf Nutzen nur noch für die Gemeinschaft und zukünftige Generationen zielt und dabei das Individuum und sein Selbstbestimmungsrecht aus dem Blick verliert.

### „Befreiung vs. Ablehnung“: das italienische Beispiel

Wenn die Rahmenbedingungen nicht oder zu spät oder zu langsam verbessert werden, entsteht das Risiko revolutionärer Entwicklungen. Deren Nutzen für die betroffenen Menschen, hier also in erster Linie psychisch Kranke und ihre Angehörigen, wird meist verfehlt, indem Geschwindigkeit und Ausmaß der intendierten Änderung die Veränderungsfähigkeit nicht nur der betroffenen Kranken, sondern vor allem auch der Angehörigen und des psychiatrischen Personals überfordern. Als Beispiel sei auf die italienische Psychiatriereform von 1978 verwiesen [10].

Unter dem Stichwort der „Befreiung“ psychisch Kranker aus den Zwängen der „totalen Institution“ psychiatrischer Anstalten wurde das Selbstbestimmungsrecht psychisch Kranker verabsolutiert. Diese revolutionäre Psychiatriereform verbesserte zwar kurzfristig die Lebenssituation einiger Patienten, ergab also einen begrenzten Nutzen, dem aber ein erheblicher Schaden gegenüberstand, indem Patienten unversorgt entlassen oder auch in nichtpsychiatrische Institutionen aufgenommen wurden, Angehörige uninformiert und überfordert waren, Verwaltungen und teilweise auch psychiatrisches Personal sich unwillig verhielten. Erst eine personalintensive sozialpsychiatrische Präsenz vor Ort ermöglichte allmählich eine Inklusion der Kranken in familiäre oder kommunale Strukturen und erreichte damit langfristig einen Nutzen der Reform für psychisch Kranke und ihre Angehörigen [11].

Fazit: Durch eine ideologisch bestimmte Instrumentalisierung [12, 13]^2^ und Schädigung von Patienten mittels einer überhasteten und technisch unzureichenden Reform wurde auch eine den Menschenrechten verpflichtete Grundidee erheblich beschädigt.

### Ausstattung vs. Stigmatisierung

Psychiatrieinterne [15] ebenso wie öffentliche Kritik [16, 17] führte nach dem letzten Krieg über die Psychiatrieenquete des Bundestages zu einer tiefgreifenden Reform, die die Rahmenbedingungen psychiatrischen Handelns erheblich verbesserte, nicht nur durch neue Gebäude und mehr und qualifiziertes Personal, sondern auch durch eine anthropologisch begründete Respektierung der Subjektivität bis hin zur Anerkennung des Selbstbestimmungsrechtes psychisch Kranker und vor allem durch die Entwicklung wirksamer Behandlungsverfahren. Der Nutzen dieser Zielvorstellungen und Möglichkeiten der zeitgenössischen Psychiatrie droht aber – wie bereits erwähnt – wieder durch Schaden verdunkelt zu werden, wenn eine angemessene Entwicklung der Rahmenbedingungen, d. h. in erster Linie eine ausreichende Zahl qualifizierten Personals, ausbleibt. Wenn beispielsweise eine 1:1-Personalbetreuung eines Patienten erforderlich ist, muss das dafür benötigte Personal von anderen Patienten abgezogen werden, muss diese also un- oder zumindest unterversorgt lassen. Hier wird nicht nur gefragt, wie gerecht solche Ressourcenverteilung ist, sondern Mitarbeiter erleben diesen Mangel auch frustriert. Denn wenn überlastetes Personal die heute möglichen und gesetzlich festgelegten Qualitätsanforderungen nicht erfüllen kann, werden enttäuschte und kritische Stimmen laut, die letztlich nicht nur die Psychiatrie, ihre Institutionen und Mitarbeiter, sondern auch die Patienten stigmatisieren. Um dem entgegenzuwirken, unterstützt die DGPPN intensiv die Petition des Bundesverbandes der Angehörigen psychisch Kranker an den Bundestag für ausreichendes Personal [18].

## Entwicklung therapeutischer Interventionen

Bisher wurden der angezielte Nutzen psychiatrischer Versorgungsstrukturen und damit verbundene Risiken angesprochen; dass aber darüber hinaus ebenso alle therapeutischen Interventionen neben dem die Indikation bestimmenden Nutzen auch Risiken enthalten und damit Stellungnahmen des Patienten erfordern, soll mit einigen weiteren Beispielen verdeutlicht werden.

So hatten die im 19. Jahrhundert herrschenden Therapieprinzipien aus heutiger Sicht ein für viele Patienten ungünstiges Nutzen-Risiko-Verhältnis; sie zeigen die therapeutische Hilflosigkeit einer vorwissenschaftlichen Psychiatrie und sind oft nur im Kontext herrschender Ideen zu verstehen, zu denen das Selbstbestimmungsrecht psychisch Kranker noch nicht gehörte.

### Isolierung

Die Isolierung des Kranken aus seiner krankmachenden sozialen Umgebung durch Aufnahme in die Anstalt wurde zeitweilig, so von einflussreichen Psychiatern wie Pinel, Esquirol, Reil zu Beginn des 19. Jahrhunderts, als ein unverzichtbares Therapieprinzip angesehen; sie nahmen zunehmend auch „sozialpsychologische Wurzeln und sozial bedingte Färbung der Psychosen“ [19] wahr, sodass angesichts der Lebensumstände der meist „armen Irren“ [4] solche Herausnahme nur zu verständlich war. Heute hingegen ist die Verminderung pathogener Spannungen im Umfeld des Kranken, z. B. durch psychoedukative Gruppen, die Regel, um ihn in seinem sozialen Feld zu halten und damit auch seine Selbstbestimmungsfähigkeit zu stärken.

### Psychische Erschütterung

Psychische Erschütterung galt als

… eine kräftige Heilmethode … den Krampf durch den Krampf brechen. Man muss psychische Erschütterungen erregen, die die Wolken, von denen die Intelligenz bedeckt ist, zerstreuen; den Schleier, der zwischen der äußeren Welt und dem Menschen schwebt, zerstören; die falsche Kette von Ideen zerbrechen … [19, S. 128].

Psychische Erschütterungen wurden durch starke Reize (z. B. Sturzbad) erzeugt, auch durch Erbrechen mittels Emetika wie Brech- und Nieswurzel (Helleborus) – oder durch Drehen in speziell konstruierten Drehmaschinen; dieser Prototyp einer mechanistischen Therapie um 1800 erscheint nicht nur heute uns, sondern erschien auch schon damals manchen Psychiatern als grausame Behandlung; sie war vorwiegend eine aversive Zwangsbehandlung. Auch wenn einzelne Patienten sich nach dem Erbrechen von „bösen Säften“ befreit fühlten, so hatte sie insgesamt doch ein ungünstiges Nutzen-Risiko-Verhältnis. Sie war eingebettet in herrschende Überzeugungen der Medizin um 1800 (Humoralpathologie, Reizlehre) und verschwand mit dem Aufkommen des „moral management“.

### „Moral management“, „no-restraint“

Das ab 1800 sich ausbreitende „moral management“ und das ihm ab Mitte des Jahrhunderts folgende „no-restraint“ begründeten einen neuen Umgang mit psychisch Kranken in den Heil- und Pflegeanstalten, indem mechanische Restriktionen und Gewalt durch eine eher psychologisch-pädagogisch orientierte Umgangsweise mit dem Patienten abgemildert oder ersetzt wurden. Ärzte suchten, mit Vertrauen und Verständnis dem Kranken zu helfen. Allerdings wurden die Patienten doch meist – der pädagogisch ausgerichteten Bildungsbeflissenheit des 19. Jahrhunderts entsprechend – eher als „unmündige“ Kinder angesehen [20, S. 1], so von Friedrich Roller, Gründungsdirektor der damaligen Modellanstalt Ilmenau und Autor des 1831 einflussreichen Buches *Die Irrenanstalt nach allen ihren Beziehungen*, dessen erster Satz lautet: „Ein Irre (!) ist unmündig wie ein Kind …“. Patienten wurden „zur Besonnenheit und zum Gehorsam genöthigt“ [21, S. 499] d. h. mittels Belohnung und Bestrafung dazu gebracht, sich der strikten Ordnung der Anstalt anzupassen. Die äußere Ordnung sollte helfen, die innere Unordnung zu überwinden.

### „Aktivere Krankenbehandlung“

Die am Beginn des 20. Jahrhunderts durch Hermann Simon eingeführte „aktivere Krankenbehandlung in der Irrenanstalt“ [22] sprach die gesunden Anteile der psychisch Kranken durch Arbeitsanforderungen an und vermochte durch den Einbezug aller Patienten die Atmosphäre der Anstalt so zu verbessern, dass sie von vielen Anstalten übernommen wurde. Durch Vorbild und Belohnung von Leistungen wurde soziale Entlassungsfähigkeit der Patienten angestrebt, aber das Risiko einer Ausbeutung von Patienten nicht immer vermieden – wenn auch die Behauptung des Genetikers Benno Müller-Hill polemisch überzogen ist, dass die psychiatrischen Anstalten mit „unbezahlter Zwangsarbeit (<Arbeitstherapie>) … den Konzentrationslagern ähneln“ [23, S. 42].

Diese rein empirisch begründeten Therapieverfahren wurden trotz ihres ungünstigen Nutzen-Risiko-Verhältnisses meist solange beibehalten, bis sie durch andere Verfahren ersetzt wurden, die zwar dem Wandel herrschender Ideen entsprechend anders begründet wurden, aber kaum ein wesentlich besseres Nutzen-Risiko-Verhältnis aufwiesen. Auch die ersten biologisch wirksamen psychiatrischen Behandlungsverfahren wie die *Malariatherapie *der progressiven Paralyse und die *Elektrokrampftherapie* der damals sog. endogenen Psychosen hatten initial ein ungünstiges Nutzen-Risiko-Verhältnis; trotzdem wurden sie jahrzehntelang angewandt, da ihre therapeutische Wirksamkeit, also ihr Nutzen, unbestreitbar war und Kontrolle der Risiken soweit gelang, dass ein akzeptables Nutzen-Risiko-Verhältnis erreicht werden konnte.

### Malariatherapie der progressiven Paralyse

Am 14.06.1917 kam dem Wiener Psychiater Julius Wagner‑v. Jauregg angesichts eines gerade eingelieferten Soldaten, der Fieberanfälle im Malaria-tertiana-Rhythmus hatte, „wie ein Blitz der Gedanke, mit dem Blut dieses Malarikers … Paralytiker zu impfen“ [24, S. 163]. Vorausgegangen war diesem Gedankenblitz eine jahrzehntelange Beschäftigung Wagner‑v. Jaureggs mit der symptomreduzierenden Wirkung von Fieber [25, 26]. Er bat deshalb einen Internisten um das Blut von Patienten, die wegen gesicherter Malaria tertiana von diesem behandelt wurden. Doch 4 der ersten 14 geimpften Paralytiker starben, da sie irrtümlicherweise Blut der gefährlichen Malaria tropica überimpft erhalten hatten [24, S. 165]. Weitere Impfungen mit kontrolliert gesichertem Malaria-tertiana-Blut verliefen dann ohne Komplikationen und erwiesen sich weiterhin als überaus wirksam gegen die progressive Paralyse [27]; damit wurde das Nutzen-Risiko-Verhältnis dieser ersten wirksamen psychiatrischen Behandlung so günstig, dass sie sich innerhalb weniger Jahre über die Welt ausbreitete und Wagner‑v. Jauregg zum Nobelpreis – dem ersten für einen klinischen Psychiater – vorgeschlagen wurde. Aber er musste 10 Jahre warten, da der schwedische Psychiater Gadelius als Mitglied des Nobelkomitees sagte, „er könne sich nicht entschließen, einem Arzt, der einem Paralytiker noch Malaria einimpfte, den Nobelpreis zu verleihen, denn er sei in seinen Augen ein Verbrecher. So mußte ich“ – fährt Wagner‑v. Jauregg in seinen Lebenserinnerungen fort – „also warten, bis dieser alte Herr pensioniert wurde“ [24, S. 169].

Hier wurde also der Nutzen einer Therapie höher bewertet als ein initial sogar tödliches Risiko. Die distanzierte Erwähnung der Todesfälle könnte auch daran denken lassen, dass im zeitgenössischen Kontext des Weltkrieges mit Millionen Toten der Tod eines Menschen anders als heute bewertet wurde. Vor allem aber konnte eine Therapie erstmals eine unheilbare chronisch progrediente Krankheit wirksam bekämpfen, eine zudem sehr häufige Krankheit, an der um die vorige Jahrhundertwende 25–50 % aller Anstaltspatienten litten und die damit ein erhebliches Versorgungsproblem darstellte [28, 29]. Schließlich konnte die dann eingeführte Sicherheit der Behandlung durch standardisierte Kontrollen schwerwiegende Komplikationen ausschließen und damit das Nutzen-Risiko-Verhältnis zugunsten des Nutzens verbessern. Erst als mit dem ab den 1940er-Jahren verfügbaren Penicillin die Neurolues noch wirksamer behandelt werden konnte, wurde die wirksame, aber aufwendige Malariatherapie eingestellt. Zwar wurde bereits in den 1920er-Jahren in den Krankengeschichten die „Zustimmung zur Behandlung von wann und von wem“ notiert [29, S. 451]; es bleibt jedoch mehr als fraglich, inwieweit die nur sehr unvollständig und grob dokumentierten Einwilligungen selbstbestimmt waren, zumal dann, wenn die Behandlung auf Wunsch von Angehörigen durchgeführt wurde; auch in jenen Fällen, in denen die Patienten die Behandlung wünschten, ist unklar, ob und wie sie über deren Nutzen und Risiken aufgeklärt wurden. So lagen z. B. in der Berliner Klinik Wiesengrund von 204 zwischen 1926 und 1941 mit Malariablut geimpften Männern nur von 142 Patienten „Einverständnis“-Erklärungen vor, davon 108 von der Ehefrau, 23 vom Patienten selbst. 12 Patienten lehnten die Malariatherapie ab und erhielten sie auch nicht [30].

Dass es sich dabei im Kontext eines noch nicht nach heutigem Verständnis ausgebildeten Selbstbestimmungsrechts in Wirklichkeit nur um einen rechtlichen Schutz des Arztes handelt, geht sehr deutlich aus einer Feststellung hervor, die unter dem Titel „Rechtliche Fragen“ des gut 20 Jahre später erschienenen Buches *Die moderne Schockbehandlung* das gesamte Thema Einwilligung in einem einzigen Absatz abhandelt:

Um sich vor ungerechtfertigten Anzeigen zu schützen, tut man gut, sich von den nächsten für den meist geschäftsunfähigen Kranken verantwortlichen Angehörigen einen Revers unterschreiben zu lassen. Der Revers soll die Form einer kurzen Zustimmungserklärung haben, in der auf die praktisch geringe Gefährdung durch Gesundheitsschädigungen aufmerksam gemacht wird. Ausführliche schriftliche oder mündliche Erläuterungen über alle denkbaren Komplikationen oder Zwischenfälle zu geben ist unnötig und im Interesse des Kranken unzweckmäßig. Die Zustimmung würde dann allzuoft verweigert werden [31, S. 14].

Hiermit ist auch das Problem angedeutet, Nutzen und Risiken der Aufklärung und Einwilligung selbst innerhalb des vorgegebenen rechtlichen Rahmens einzuschätzen (s. a. Abschnitt „Psychotherapie“).

### Elektrokrampftherapie

Ähnlich wie die Malariatherapie wurde die Elektrokrampftherapie (EKT) wegen ihrer Wirksamkeit gegen schizophrene und affektive Psychosen trotz anfänglich erheblicher Risiken ab 1938 bis in die 1960er-Jahre weltweit eingesetzt, nachdem grobe körperliche Komplikationen wie z. B. Frakturen durch entsprechend modifizierte Anästhesien vermieden werden konnten. Allerdings stimulierten diese initialen Nebenwirkungen eine anhaltende demagogische Kampagne gegen die EKT; dies führte einerseits dazu, dass diese Behandlung eine der zunächst wenigen war, zu denen Psychiater eine schriftliche Einwilligung der Patienten einholten; andererseits waren viele Psychiater auch erleichtert, mit dem Aufkommen der anscheinend leichter zu handhabenden Psychopharmakotherapie auf die EKT verzichten zu können. Kenner der EKT sehen in ihrem völligen Verzicht jedoch ein ethisches Problem, wenn es als lebensrettende Methode der Wahl, so bei therapieresistenten Depressionen, febrilen Katatonien oder dem malignen neuroleptischen Syndrom, nicht mehr zur Verfügung steht [32–37]. Abgesehen von einer skeptischen Sicht auf die Wirksamkeit der EKT bezieht sich eine aktuellere Diskussion auf Umfang und Modus der Aufklärung [38] und Gültigkeit der Einwilligung [39, 40].

### Psychiatrische Pharmakotherapie

Dass jedoch auch die die EKT ablösende psychiatrische Pharmakotherapie nicht nur Nutzen, sondern ebenfalls inzwischen gut bekannte Risiken birgt und damit auch diese Therapie einer – und zudem nicht nur einmaligen – Nutzen-Risiko-Abwägung bedarf, soll hier nur kurz skizziert werden (s. a. Abschnitt „Psychotherapie“).

Die Entwicklung wirksamer Arzneimittel in den 1950er-Jahren veränderte die Atmosphäre psychiatrischer Kliniken bedeutsam. Therapeutische Aufbruchsstimmung beherrschte junge Psychiater. Akute psychotische Erregungen konnten schnell unter Kontrolle gebracht werden, sodass viele Patienten relativ bald wieder entlassen bzw. längere Liegezeiten vermieden werden konnten. Ebenso konnte eine ambulante medikamentöse Rezidivprophylaxe Krankenhausaufnahmen vermeiden helfen. Somit konnten auch die sozialen Bezüge der Patienten differenzierter erkannt werden und für die Patienten eher erhalten bleiben [41]. Solch Nutzen für die Patienten wurde jedoch durch allmählich wahrgenommene unerwünschte Wirkungen der sog. Psychopharmaka überdeckt; darunter leidende Patienten wurden non-compliant und begannen zu protestieren, verteufelten schließlich die psychopharmakologische Therapie als „chemische Knebel“ [42]. Schädliche Folgen waren Krankheitsrezidive durch einerseits unkontrollierte Beendigung einer wirksamen Therapie („discontinuation syndrome“; [43–45]), andererseits aber auch durch Ablehnung indizierter Medikationen. Diesen negativen Wirkungen der Psychopharmakotherapie wird begegnet durch eingehende Aufklärung des Patienten über Nutzen und Risiken der Medikation, heute auch einschließlich von Vor- und Nachteilen eines Absetzens sowie stärkerer Berücksichtigung seiner Wünsche oder auch durch eine fraktionierte Aufklärung; wichtig ist besonders in diesen Fällen auch die psychoedukative Einbeziehung von Angehörigen. So muss beispielsweise über harmlose Frühdyskinesien vor Beginn einer antipsychotischen Medikation aufgeklärt werden, da sie einen nicht aufgeklärten Patienten stark ängstigen und damit die ganze therapeutische Bemühung infrage stellen können, während über das schwerwiegende Risiko einer Späthyperkinese erst vor dem Übergang auf eine antipsychotische Erhaltungsmedikation aufgeklärt werden muss, da dieses Risiko frühestens nach 3 Monaten eintritt [46–48]. Oder wenn man rezidivgefährdeten Patienten erklärt, warum bei ihnen eine rezidiv-prophylaktische Langzeitmedikation unverzichtbar sei, sollte eine trotzdem erfolgende Ablehnung mit der Zusage kommentiert werden, dass man selbstverständlich bereit ist, den Patienten bei einem erneuten Rezidiv, das wegen fehlender Medikation sehr wahrscheinlich sei, wieder in Behandlung zu nehmen; so konnten sich manche Patienten erst – aber doch immerhin – nach dem zweiten oder dritten Rückfall zur medikamentösen Rezidivprophylaxe entscheiden. Diese Akzeptanz des Selbstbestimmungsrechtes mag zwar das Vertrauen des Patienten fördern; dass aber solcher therapeutischer Langmut, der das hohe Rezidivrisiko trotz vorhandener therapeutischer Möglichkeit nicht vermindert, auch schwerfällt, ist angesichts hoher sozialer und finanzieller Kosten eines Rückfalls wohl verständlich.

Vergleicht man das Fehlen jeglicher Hinweise auf die Einwilligung von Patienten in eine versuchsweise Anwendung des ersten Antipsychotikums Megaphen 1953 [49] mit der heute selbstverständlichen Aufklärung vor jeder Psychopharmakotherapie, dann wird ein Wandel der ärztlichen Einstellung zum Patienten deutlich, dessen Kontext wenigstens angedeutet werden soll: 1966 ergab eine Analyse publizierter klinisch-wissenschaftlicher Untersuchungen, dass Versuchspersonen in der Erprobung neuer medikamentöser Verfahren in der Regel nicht, insbesondere nicht über Risiken aufgeklärt worden waren [50]. Nicht zuletzt die Prominenz des Autors, Inhaber des weltweit ersten Lehrstuhles für Anästhesie an der Harvard Medical School, provozierte mit dieser Arbeit die bioethische Diskussion in den USA. Das Konzept der Einwilligung nach Aufklärung, der „informed consent“, wurde zunächst für die klinische Forschung entwickelt, dann aber auf jegliche Therapie übertragen und heute auf jede patientenbezogene ärztliche Handlung ausgedehnt [51]. Dass die damit verbundene, in Deutschland bereits 1900 und erst recht seit dem Nürnberger Kodex von 1949 rechtlich geforderte Respektierung des Selbstbestimmungsrechtes des Patienten sich jedoch erst in diesem Jahrhundert in der ärztlichen Praxis durchzusetzen beginnt, zeigt wie schwierig dieser Wandel von einer paternalistischen zu einer partizipativen Einstellung des Arztes ist, der den akut Kranken als hilfs- und unmittelbar handlungsbedürftig erlebt und erst mit der Erfahrung der zunehmenden Chronizität von Krankheiten auch den leidenden Kranken, seine Bewältigungsversuche, seine Einschränkungen und Fähigkeiten kennenlernt. Mit diesem Übergang von der erfolgreichen Akutmedizin zur Langzeitmedizin werden auch die rechtlichen Vorgaben für das ärztliche Handeln eingängiger und das Selbstbestimmungsrecht des Patienten stärker wahrgenommen.

### Psychotherapie

Auch psychotherapeutische Interventionen bewirken nicht nur erwünschte Veränderungen, sondern können ebenso zu unerwünschten Wirkungen führen. Deshalb soll hier auf das Problem der Aufklärung in der Psychotherapie fokussiert werden. Auch Psychotherapeuten müssen ihre Patienten über Nutzen und Risiken aufklären. Aber nicht immer ist klar, was Nutzen und was Risiken sind. Abgesehen von der persönlichen Überzeugung des Psychotherapeuten blieb der wissenschaftliche Nachweis, dass Psychotherapie nützlich sei, lange ungewiss. Dies hing auch damit zusammen, dass recht unterschiedliche Erfolgskriterien benutzt wurden; so definierten Verhaltenstherapeuten den Nutzen als Abnahme und Verschwinden störender Symptome und das Auftreten neuer Symptome als Schaden, während Psychoanalytiker etwa die Entwicklung depressiver Symptome im Behandlungsverlauf als Zeichen einer erfolgreichen Auseinandersetzung des Patienten mit seiner abgewehrten Depressivität ansahen. Und Risiken von Psychotherapie wurden – wenn überhaupt – meist als gering eingeschätzt [52, S. 43]. Erst in den letzten Jahren beginnt eine systematische Untersuchung der Risiken von Psychotherapie [53, 54]; so sind „therapieunabhängige Negativereignisse, etwa durch äußere Einwirkungen oder durch den eigengesetzlichen Verlauf der Krankheit, zu trennen von therapiebedingten Nebenwirkungen und von Therapie- bzw. Therapeutenfehlern“ [55, S. 54]. Nicht immer sind unerwünschte Ereignisse eindeutig als Wirkung oder Nebenwirkung zu klassifizieren, etwa die Auflösung einer pathogenen Partnerbeziehung oder die passagere Verstärkung einer Depression. Aber nicht nur diese Unschärfen sind Grund für unzureichende Aufklärungsraten; denn auch das Nutzen-Risiko-Verhältnis der Aufklärung selbst kann etwa bei ambivalenten oder suizidalen Patienten Psychotherapeuten veranlassen, eine Aufklärung zurückzustellen; schließlich werden auch einzelne Behandlungsschritte im Verlauf des psychotherapeutischen Behandlungsprozesses erläutert, ohne dass sie vermutlich bewusst den Aufklärungserfordernissen zugeordnet werden; man kann hier von impliziter Aufklärung sprechen.

## Fazit für die Praxis

Jede psychiatrische Intervention birgt neben dem intendierten Nutzen auch Risiken. Deshalb ist eine sorgfältige Nutzen-Risiko-Abwägung vor jeder Intervention unter kompetenter Kenntnis auch der potenziellen kurz- wie langfristigen Risiken erforderlich. Diese Abwägung sollte bei unerwünschten Ereignissen und auch bei längerem Verlauf wiederholt werden, da sich das Nutzen-Risiko-Verhältnis sowohl im individuellen Fall wie auch für ein bestimmtes Therapieverfahren verschieben kann: oft in Richtung vermehrter Risiken, seltener zum Nutzen. Über das Nutzen-Risiko-Verhältnis soll der Patient so informiert werden, dass er zu einer selbstbestimmten Entscheidung kommen kann. Dabei sind Einseitigkeiten oder gar dogmatische Behauptungen zu vermeiden.

## Footnotes

1 „§ 32. Diejenigen, welche wegen noch nicht erlangter Volljährigkeit, oder wegen eines Mangels an Seelenkräften, ihre Angelegenheiten nicht selbst gehörig wahrnehmen können (§. 25–31.), stehen unter der besondern Aufsicht und Vorsorge des Staats“.

2 Als ideologisches Fernziel auch des Heidelberger Sozialistischen Patienten-Kollektivs (SPK), das personelle Verbindungen zu den italienischen Psychiatrie-Reformern um Franco Basaglia hatte, nennt Heinz Häfner „die revolutionäre Beseitigung des Herrschaftssystems, der Medizin und des politischen Systems der Bundesrepublik“ [14].
